# Emotional Exhaustion and Health Complaints as Indicators of Occupational Diseases Among Civil Servants in Spain

**DOI:** 10.3390/jcm7120523

**Published:** 2018-12-07

**Authors:** Gabriela Topa, José F. Jurado-Del Pozo

**Affiliations:** 1 Department of Social and Organizational Psychology, Faculty of Psychology, National Distance Education University (UNED), 28040 Madrid, Spain; 2 Health Psychology Program, International School of Doctorate, National Distance Education University (UNED), 28015 Madrid, Spain; jjurado86@alumno.uned.es

**Keywords:** occupational diseases, burnout, stress, emotional exhaustion, health complaints

## Abstract

Occupations focused on helping others, guaranteeing people’s security, and defending societal values can be stressful and risky for the workers involved. Emotional exhaustion and health complaints usually emerge as indicators of the stress suffered by these workers in undertaking their roles. This research aims to explore the effects of psychological contract breach on both emotional exhaustion and health complaints among three kinds of Spanish workers, namely firefighters (*n* = 80), professional soldiers (*n* = 77), and prison officers (*n* = 107). The predictor variables include job tenure and psychological contract breach. Criterion variables are employees’ emotional exhaustion and health complaints. Our findings showed that job tenure and contract breaches significantly explain both emotional exhaustion and health complaints. Despite these general findings, some differences emerge as a function of the organizations. Significance levels were higher for prison officers than for firemen and Spanish soldiers. Results are discussed, suggesting conceptual integration and direction for future risk prevention in this kind of public organization.

## 1. Introduction

Some occupations are highly relevant in modern societies because of the wide range of services (such as health, education, and security) that they provide for the general population [1,2]. Although there is some debate about distinctive characteristics, most authors agree on the fact that certain occupations can be dangerous for workers, for example firefighters, prison officers, or professional soldiers [3]. Among other features, time pressure, risky duties, being in contact with demanding citizens, and working in strictly regulated environments such as prisons can be a source of additional stress for these professionals. One response to occupational stress is health complaints. While these health complaints are not diseases as such, they may still negatively influence employees’ performance and participation in working life and social events. Moreover, they are some of the main causes of long-term sick leave in European countries [4]. 

In Spain, beyond the stressful features of the activities themselves, firefighters, prison officers, and professional soldiers are workers embedded in public firms [5]. As different authors recognize, public firms are very complex organizations, aimed at producing and/or providing a broad array of products and services to society, with, among others, the peculiarity that the State is responsible for their management [6]. This fact entails a shared occupational environment for these workers, with some characteristics that can be an additional source of stress for them.

On the one hand, during the recent economic crisis, some restructuring (often accompanied by substantial downsizing) has been conducted in these organizations, exerting a potential negative pressure on employee’s well-being [7]. Moreover, these are hierarchical public organizations that belong to the State Security Forces, and they are subject to strict legal regulation in the performance of their tasks. On the other hand, a common element among these firms is the relevance of the worker’s risk prevention as a way of ensuring employee’s well-being [8]. Among others, prevention of occupational is a key tool to retain some of the main assets of the firm: its employees [9,10]. Many empirical studies have shown the predictive power of job satisfaction and psychological well-being with respect to a broad array of desirable outcomes, both for the person and the organization [11]. Summing up, the key to the success of this kind of public organization seems to depend on employee physical and psychological well-being. However, people’s attitudes and behavior in organizations partially depend on their perceptions and valuations of the relevant dimensions of the firm. Therefore, the present study will analyze the factors that predict physical and psychological well-being in a sample of Spanish public employees, namely firefighters, prison officers, and professional soldiers, who develop their activity under high-risk conditions.

### 1.1. Public Employees’ Wellbeing and Psychological Contract Breach

The psychological contract is understood as a series of individual beliefs referring to the terms of an exchange agreement between a person and another part, in this case, between the employee and the firm [12]. This contract, which exceeds the merely written agreement between two parts [13], allows them to define a series of duties and obligations to which both parts are committed within the context of that occupational relation [14]. It is based on a series of promises—implicit or explicit—that both parts exchange throughout the process of recruitment and selection, incorporation into the firm, and development of the occupational relation. With the passing of time, one of the parts, generally the employee, may develop the perception that the firm has not fulfilled its promises and obligations towards him. This perception of psychological contract breach is directly followed by an increase in undesirable outcomes such as burnout, emotional exhaustion, absenteeism, and finally departure from the job and the organization, and at the same time, a decrease in desirable outcomes, such as job satisfaction, commitment to the firm, and organizational citizenship behaviors. In fact, in recent years, a large body of empirical research has accumulated that supports these relations [15]. 

Nevertheless, most of the studies have focused on employees of private firms and there are very few empirical works analyzing these relations in the public sphere [16]. Moreover, there is a lack of research on the specific impact of perception of breach among those workers developing high risky activities, who usually put under threat their own security to protect others [17]. Besides the lack of empirical studies on public employees’ psychological contracts, some misconceptions about these professionals are widespread. For instance, early retirement options, high employment security, or high salaries can be viewed as privileged working conditions [18]. The situation of these professionals in Spain, as employees in public firms, offers a series of special characteristics since, at least in most European countries, many of them hold the category of a public civil servant. Even though this situation could be considered an enviable panorama for many other workers, these employees also frequently perceive that the public firms have broken the promises made at the time of their incorporation. 

Specifically, to date, few empirical works have been published analyzing the results of the perception of contract breach among public employees in the European setting and even less so with respect to these specific risky professional activities [18]. The empirical data provided by Vincent Cassar [16] in his investigations, verify the pattern, with a relationship between perceived contract breach, on the one hand, and a decrease in employee wellbeing, on the other. 

### 1.2. Risky Occupations and Emotional Exhaustion

Burnout, which was considered a response to chronic occupational stress, includes three components: high emotional exhaustion, low feelings of professional efficacy, and high levels of cynicism towards the task performed. Currently, however, diverse theoretical approaches consider emotional exhaustion as the core of the syndrome and propose that cynicism is rather a worker’s coping strategy in work situations where he feels he must redefine his relationships with the client and with his employers [19]. 

Few studies have empirically explored the relations between the perception of psychological contract breach and burnout. One investigation [20] showed the influence of perceived breach on emotional exhaustion and lack of job satisfaction in the employees of a financial corporation, whereas another study found similar results in Portuguese soldiers regarding burnout and engagement [18]. 

Based on the reviewed studies, this work will analyze the relations between the perception of psychological contract breach and employees’ emotional exhaustion, on the one hand, and health complaints, on the other. In the present study, we hypothesize that employees’ perception of psychological contract breach will be positively related to their emotional exhaustion (Hypothesis 1) and that the employees’ perception of psychological contract breach will be positively related to their health complaints (Hypothesis 2). 

Moreover, in most of the public organizations, job tenure can be considered a variable with explanatory power over job satisfaction and the indicators of personal well-being. And this has a double meaning. Firstly, job tenure may be considered a proxy for the position in the organizational hierarchy, while favoring workers’ development of adaptation strategies to job demands and occupational stressors. Consequently, in the present study, we hypothesize that job tenure will be negatively related to emotional exhaustion (Hypothesis 3). Secondly, job tenure will positively correlate with age, and older workers can be expected to have more health complaints. Therefore, in the present study, we hypothesize that job tenure will be positively related to employees’ health complaints (Hypothesis 4). Since the kind of duties and job design are slightly different among the three organizations, the length of the workday that the employees devote to the services’ recipients can affect the results. Hence, the percentage of the workday invested in this kind of activities will be included as a control variable. 

## 2. Method

### 2.1. Sample and Procedure 

The present study was carried out with three samples of Spanish public employees. The first sample includes firemen (*n* = 80), the second includes professional soldiers of the Spanish army (*n* = 77), and the third includes prison officers (*n* = 107). Agencies were in five different administrative regions. The Ethical Committee of the National Distance Education University (UNED) approved the study design and collection procedures. Data collection was done by means of questionnaires with diverse scales that were distributed to the participants, who completed them anonymously and handed them into the collaborators of the research team. Beforehand, the participants were informed verbally about the purpose of the investigation and they gave their written consent to survey and to the global treatment of the data. 

Among the sociodemographic variables, the mean age of the sample revealed differences between the three organizations. While the mean age for firemen was 40.44 years (*SD* = 8.53) and for prison officers, it was 38.3 years (*SD* = 7.2), professional soldiers were younger (mean age 28.43 years, *SD* = 7.9). Overall, 61.5% of the participants were male. Regarding the percentage of the workday the workers spent in contact with the recipients of their services—clients, patients, prisoners, or victims, 64.1% spent at least 50% of their day, whereas the rest spent most of their workday dedicated to these tasks. 

### 2.2. Instruments 

The dependent variables were emotional exhaustion and subjective health complaints’. The predictor variables were perceived psychological contract breach and job tenure in the organization and we controlled for the influence of percentage of workday dedicated to attending the recipients of the service. To operationalize the variables, the following instruments were used.

- *Perceived psychological contract breach*: This scale, designed for this investigation, has three items of global ratings of the degree of promise fulfillment by the organization, like those used in prior studies [21]. Some of the statements are: “I get angry when I think about what I give and what I receive from the institution where I work” or “My firm does not fulfill its commitments to me at all”. Despite the scarce length of the scale, its reliability, as assessed by Cronbach’s alpha, was α = 0.77.
- *Emotional exhaustion*: The *Cuestionario Breve de Burnout* (CBB) (The Brief Burnout Questionnaire) of Moreno et al. [22] was used to assess emotional exhaustion. The original version of this instrument has 21 items that include antecedents and consequences of the syndrome, but not all of them were used in the final data analysis. The global reliability of the scale finally used in our study was α = 0.77. The Cronbach’s alpha reliability of the six items that make up the subscale of Emotional Exhaustion was α = 0.81. Some example items are: “The demands and behaviors of the people I attend irritate me”; “My work is repetitive”; “My work is boring”; and “In general, I am tired of my work”. Agreement with the items was rated on a 5-point Likert scale ranging from 1 (strongly disagree) to 5 (strongly agree).
- *Health complaints*: The reduced Spanish version of the Subjective Health Complaints Inventory (SHC), created by Eriksen et al. [23], was used to assess possible employees’ health problems. Self-reported health complaints were measured by means of 10 items with rating scales ranging from 1 (never) to 5 (always), which can be categorized into two subscales: pseudo-neurology (tachycardia, sleep problems, tiredness, dizziness, anxiety, and sadness/depression), and gastrointestinal problems (heartburn, ulcer, stomach ache, and diarrhea). Despite the briefness of the scale, its reliability, as assessed through Cronbach’s alpha, was α = 0.69.

Job tenure was measured by asking participants how many years they had worked in the present job, and workday was measured as a percentage (ranging from 0 to 100) of the workday spent in contact with prisoners, clients or victims. 

## 3. Results 

To test the hypotheses of the study, we first performed bivariate correlational analysis (Pearson product-moment correlations) among the variables and analysis of variance as a function of membership in the diverse organizations. Secondly, we conducted multiple linear regression analysis of the predictor variable separately on the different criteria, controlling for the influence of workday contact with the service recipients. The relations among the target variables were analyzed for the global sample and for each organization, enabling us to refine the explanatory hypotheses of the study.

The descriptive statistics of the variables and the correlation matrix are presented in Table 1, which shows that the mean of psychological contract breach is high, exceeding the theoretical mean of the scale. The value of employee health complaints is close to the theoretical mean, and emotional exhaustion is lower than the theoretical mean. Bivariate correlations are an initial approach to the relationship between the study variables, revealing that a contract breach is positively related to emotional exhaustion and health complaints, and all the values are statistically significant. 

We used analysis of variance, with membership in different organizations as the predictor to detect possible significant differences among the organizations in the target variables. The analysis of variance was significant for contract breach (*F* (2, 210) = 4.58, *p* < 0.01), for emotional exhaustion (*F* (2, 259) = 35.16, *p* < 0.001) and for health complaints (*F* (2, 259) = 17.798, *p* < 0.001).

**Figure 1:**
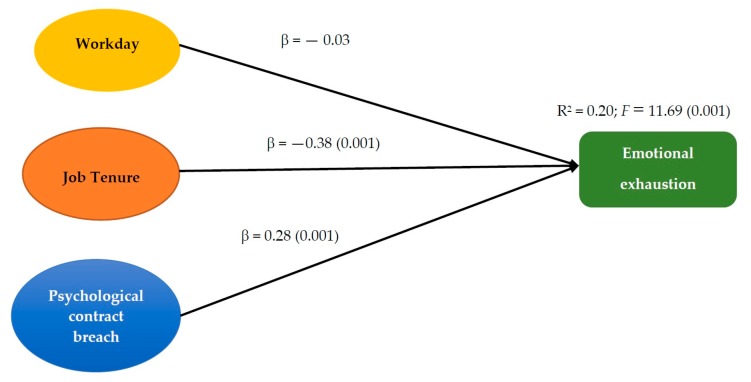
Results of the regression analysis on emotional exhaustion.

**Figure 2:**
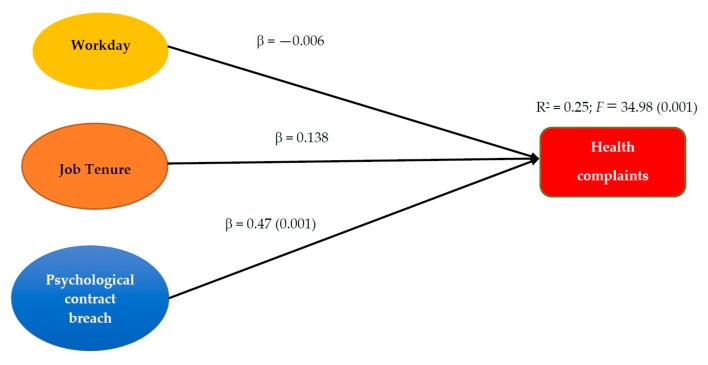
Results of the regression analysis of health complaints.

Multiple linear regression analysis was used to test two models: the first one incorporated workday, to control for their effects on the dependent variables, and the first predictor variable job tenure. In the second model, we added the other predictor variable psychological contract breach. This analysis was conducted first with the global sample and then with each organization separately. Figure 1 and Figure 2 present the standardized regression weights and the percentage of explained variance for each criterion variable in the global sample, and Table 2 for the subsamples. 

Job tenure in the organization had a significant and negative regression weight in the prediction of emotional exhaustion, indicating that the newest staff members suffered the greatest emotional exhaustion. The model improved notably when adding psychological contract breach, which is an efficacious predictor, as it explained 20% of the variance of emotional exhaustion. When applying the same procedure to each subsample, all the results point in the hypothesized direction, because contract breach was a statistically significant predictor for firemen, soldiers, and prison officers. Nevertheless, the model with contract breach explained nearly 30% of the variance of emotional exhaustion among soldiers and prison offers, but less so with the firemen sample. 

In the prediction of health complaints, job tenure in the organization had predictive power in the first model, but when adding contract breach, its standardized regression weight dropped significantly. Contract breach, in contrast, had a considerable impact on employees’ health complaints, predicting 25% of the total variance in the regression model. In this case, the regression Model was considered more adequate for the samples of firemen and prison officers, where it explained more than 20% of the variance of health complaints, but this percentage dropped to 14% among the professional soldiers. Summing up, we can state that this study found support for the proposed hypotheses. 

## 4. Discussion 

The present study analyzed the incidence of certain psychosocial variables, such as the psychological contract breach, on the workers’ perceived outcomes in a sample of Spanish public employees. These results corroborate and extend prior findings. Firstly, we verified the impact of psychological contract breach on undesirable outcomes such as employees’ emotional exhaustion and health complaints. Secondly, previous findings are extended, revealing that this pattern of relations is maintained even in work spheres that are, to some extent, protected by their occupational stability, their high degree of syndication, and the scarce presence of human resource management practices that are typical of private firms. 

Specifically, it is interesting to observe that on emotional exhaustion, both job tenure and contract breach showed similar statistical power, whereas the effect was the opposite direction. In this sense, although the present study did not assess this aspect, one could speculate that employees who had worked longer in the institutions had developed more effective coping strategies for their workplace stressors and were, to some extent, safe from them. Accordingly, various authors have indicated that professional cynicism—understood as a critical and mistrusting attitude towards the utility of their task at the mid and long-term—is a coping strategy of workers in situations of prolonged stress. In contrast, when assessing employees’ health complaints, the data support the importance of contract breach and the reduced importance of job tenure in the post. This finding again reveals that the soft factors, although more difficult to conceptualize and measure operationally, have a great impact on outcomes at the personal, group, and organizational levels. 

Considering the different organizations to which the employees belonged, the analyses conducted reveal the differences among them. Although the activities carried out by these groups of workers are similar—most of them attend to people in emergency situations—the differences found may be due to the existence of specific organizational variables, either associated with specific task characteristics or with the defining traits of the diverse organizational cultures. Other studies have explored the influence of organizational cultures like those considered herein on relevant outcomes at the personal and organizational level, such as job satisfaction and organizational commitment among the positive outcomes, and mobbing among the negative ones [24,25].

In general, it can be stated that psychological contract breach is a relevant variable to explain undesirable outcomes of public sector employees. These relations make sense when considering that contract breach, since it consists of the perception of breaking the promises made when the worker joined the organization, can imply the employee’s loss of control of the situation, first leading to feelings of disorientation and subsequently to feel anger or betrayal. In this sense, it would also involve an increase in uncertainty and, thus, we can better understand its potential value as a stressor, even in work settings that seem more stable and less subject to change, like the ones studied herein.

Future studies should explore the prior health status of the participants, based on organizational records because controlling their influence on later health complaints can provide stronger conclusions. 

From the viewpoint of intervention, we think that this study can contribute relevant information with a view to improvement in human resources management of public firms. Currently, psychological contracts have changed and evolved towards individual agreements between employers and workers, especially when concerning knowledge professionals [26]. This implies that the managers could implement some measures to guarantee more stable psychological contracts. Among other recommendations, we suggest forming personnel selectors, so they can offer future candidates realistic information during the recruitment process, providing a specific description of the work to be carried out in the organization. In this process, it might be appropriate to draft the result of the negotiations in some document that would describe in detail the agreements reached by both parts. It would also be suitable to establish orientation programs for new employees: these programs would be a series of regulated steps to help the new workers reduce their uncertainty about their role in the firm and build the meaning of the new situation, as suggested by Sutton and Griffin [27]. Moreover, as these occupations are loaded with psychological effects resulting from their duties, provision of preventive psychological support by the employer can be introduced in the public settings, as the EU Occupational Safety and Health Strategic Framework 2014–2020 strongly recommends. From the primary prevention point of view, actions oriented to strengthen personal resources for stress coping, as resilience [28] or size, strength and diversity of social networks [29] could serve as efficient anticipatory interventions [30]. 

In this vein, employees that longer remain in these organizations usually experienced a re-socialization process when they advance in their careers or change from one department from another. These events mean a review of the psychological contract’s agreements, and they can result in a new breach. Hence, human resources departments should be very vigilant on the changing work conditions that can negatively affect employees’ wellbeing. At the same time, our findings have strong implications for workers. Each employee must be aware of his or her own responsibility for the management of the professional career. Hence, the development of his or her intrapreneurial self-capital can be a promissory individual resource for coping the ongoing changes in the work environment [31]. 

## 5. Conclusions

Despite the great difficulty to access this type of organizations, we think that the results can contribute to improving the human resources management of public firms, as well as prevention and treatment of occupational diseases [32]. 

## Figures and Tables

**Table 1:** Descriptive statistics and correlation matrix for each sample.

	Firemen (*n* = 80)						Professional Soldiers (*n* = 77)						Prison Officers (*n* = 107)					
**Variables**	**Mean**	**SD**	**1.**	**2.**	**3.**	**4.**	**Mean**	**SD**	**1.**	**2.**	**3.**	**4.**	**Mean**	**SD**	**1.**	**2.**	**3.**	**4.**
1. Workday (percentage of day)	63	12	1				39	10	1				83	9	1			
2. Job tenure (years)	14.3	8.1	−0.18	1			7.34	6.09	−0.42 *	1			12.3	6.9	−0.15	1		
3. Psychological contract breach	3.56	0.95	0.20	−0.06	1		3.24	0.88	0.46 *	−0.31	1		3.01	0.81	−0.16	0.11	1	
4. Emotional exhaustion	2.24	0.67	−0.09	0.30 **	0.24 *	1	2.87	0.81	−0.30 *	0.009	0.11	1	3.2	0.76	−0.06	0.11	0.41 **	1
5. Health complaints	3.45	0.85	−0.02	−0.10	0.36 **	0.40 **	2.55	1.12	0.13	0.10	0.14	0.38 **	3.05	0.86	0.06	0.10	0.34 **	0.52 **

* *p* < 0.05. ** *p* < 0.01.

**Table 2:** Predictors of emotional exhaustion and health complaints in each sample.

	Standardized Regression Coefficients		
	Professional Soldiers	Prison Officers	Firemen
**Predictor Variables**	**Criterion variable: Emotional exhaustion**		
Workday	-	0.04	−0.11
Job tenure	−0.12	0.00	0.25
Psychological contract breach	0.33 (0.05)	0.52 (0.001)	0.31 (0.05)
*F*	5.24 (0.05)	25.33 (0.001)	5.40 (0.05)
*R* ^2^	0.12	0.28	0.17
**Predictor Variables**	**Criterion variable: Health complaints**		
Workday	-	0.13	−0.16
Job tenure	−0.10	0.05	−0.13
Psychological contract breach	0.37 (0.05)	0.50 (0.001)	0.42 (0.01)
*F*	6.72 (0.05)	22.48 (0.001)	10.24 (0.01)
*R* ^2^	0.14	0.25	0.17

*p* values in brackets.
